# Myo‐inositol oxygenase (MIOX) accelerated inflammation in the model of infection‐induced cardiac dysfunction by NLRP3 inflammasome

**DOI:** 10.1002/iid3.829

**Published:** 2023-05-05

**Authors:** Wenjun Zhou, Congyi Yu, Yiwen Long

**Affiliations:** ^1^ Department of Critical Care Medicine, Ruijin Hospital, Lu Wan Branch Shanghai Jiaotong University School of Medicine Shanghai China

**Keywords:** cardiac dysfunction, infected, MIOX, NLRP3, ubiquitination

## Abstract

**Background:**

Cardiac dysfunction is an important component of multiple organ failure caused by sepsis, and an important cause of high mortality in patients with sepsis. Herein, we attempted to determine whether myo‐inositol oxygenase (MIOX) has proinflammation enzyme in infection‐induced cardiac dysfunction (IICD) and its underlying mechanism.

**Methods:**

Patients with IICD were collected by our hospital. A mouse model of IICD was induced into male db/db mice by cecal ligation and puncture (CLP). All mice were injected with 20 μL of LV‐MIOX or LV‐control short hairpin RNA using a 0.5‐mL insulin syringe. On the second day, all mice were induced by CLP. H9C2 cell was also induced with lipopolysaccharide and adenosine triphosphate. Quantitative analysis of messenger RNAs (mRNAs) and gene microarray hybridization was used to analyze the mRNA expression levels. Enzyme‐linked immunosorbent assay, immunofluorescence, and Western blot analysis were used to analyze the protein expression levels.

**Results:**

The serum expressions of MIOX mRNA level in patients with IICD were upregulated compared to normal healthy volunteers. MIOX promoted inflammation levels in the in vitro model of IICD. Si‐MIOX inhibited inflammation levels in the in vitro model of IICD. MIOX accelerated inflammation and cardiac dysfunction in infection‐induced mice. MIOX interacted with NLR family pyrin domain containing 3 (NLRP3) protein to reduce the degradation of NLRP3. The inhibition of MIOX reversed the effects of NLRP3 in the in vitro model of cardiac dysfunction.

**Conclusions:**

Taken together, these findings demonstrate that MIOX accelerates inflammation in the model of IICD, which may be, at least in part, attributable to NLRP3 activity by the suppression of NLRP3 degradation in IICD.

## Graphical abstract

Myo‐inositol oxygenase accelerated inflammation in the model of infection‐induced cardiac dysfunction, which may be, at least in part, attributable to the induction of NLR family pyrin domain containing 3 (NLRP3) inflammasome through the inhibition of NLRP3 degradation in infection‐induced cardiac dysfunction.

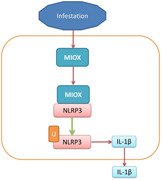

## 1 INTRODUCTION

According to statistics, in 2013, the number of deaths due to cardiovascular diseases exceeded 17.3 million globally, and the incidence of cardiovascular diseases increased by 41% compared with the past two decades.^1^, ^2^ The number of sepsis patients exceeded 20 million each year, and the deaths of hospitalized patients due to infection was about 5.3 million.^3^ Severe sepsis is a complex syndrome characterized by one or more organ dysfunction (especially cardiac dysfunction) and hemodynamic disorders. Sepsis‐induced cardiac dysfunction mainly refers to the decline of left ventricular contractility. Epidemiological survey shows that the mortality rate of patients with sepsis is as high as 20.6%, and 79 of every 100,000 people die of sepsis every year.^2^ The mortality rate of septic shock patients is generally more than 40%, even up to 70%. However, in patients with sepsis, the unregulated sympathetic tension has stimulated researchers to study selective inhibition of sympathetic tension, which potentially further continues this sympathetic dysfunction.^3^

The main reason for sepsis, leading to a high incidence rate and mortality, is the dysfunction of the cardiovascular system.^2^ Systemic infection (sepsis) has recently been defined as “fatal organ dysfunction caused by an infection‐induced and dysfunctional host defense response.” Because it is closely related to mortality, the new definition emphasizes the importance of sequential organ dysfunction after infection and the clinical significance of timely diagnosis.^3^ More than 30 years of research have found that changes in the function of the circulatory system and development of organ function damage caused by systemic infection. For example, the decline of peripheral vascular resistance and the low response to catecholamine is the pathophysiological basis of shock. The changes in capillary permeability affect the preload, cause tissue edema, aggravate tissue perfusion disorder, and closely participate in the process of shock. However, cardiac dysfunction (cardiac systolic and diastolic dysfunction) will affect cardiac output and further lead to hemodynamic deterioration.^3^

Sepsis involves multiple molecular mechanisms such as infection, microcirculatory and mitochondrial disorders, enterogenous endotoxemia and bacterial translocation, inflammation and immune disorders, coagulation dysfunction, and oxidative stress imbalance.^4^, ^5^ It is a common acute critical disease in clinical practice. It is characterized by a high incidence rate, high mortality, rapid disease progression, and high complications, which are very likely to lead to the occurrence of multiple organ dysfunction. Therefore, clarifying the pathogenesis of sepsis and formulating appropriate treatment strategies is an important basis for reducing the mortality of sepsis.^4^, ^5^

Severe infections are generally accompanied by compensatory low cardiac output and tachycardia, which can increase the patient's heart burden and myocardial oxygen consumption, affect coronary blood perfusion, and so on, and induce cardiac insufficiency.^4^, ^5^ Although early goal‐oriented treatment can control infection, inhibit the inflammatory response, and relieve symptoms, many patients still have persistent arrhythmia and mildly low cardiac output, which might be related to sympathetic overexcitement.^6^ Moderate inflammatory reaction is beneficial for the body to resist infection. However, excessive activation or prolonged inflammatory reaction is often harmful to the body. The sympathetic nerve tension caused by sepsis is too high, and the disorder of inflammatory reaction is directly related to the dysfunction of myocardial cells. The original observation from animal experiments has carried out extensive research on specific “myocardial inhibitory factors,” proving that cytokines and p38 mitogen‐activated protein kinase pathway), high catecholamine cytotoxicity, complement system, nitric oxide regulation disorder, oxidative stress of myocardial cells, and so on.^4^, ^5^ At the cellular level, increased proteolysis, mitochondrial damage, nitric oxide imbalance, beta‐adrenergic receptor downregulation, and calcium imbalance can all induce cardiac dysfunction during sepsis. Cardiac dysfunction is a harmful complication of patients with sepsis, with a mortality rate of up to 70%.^4^, ^5^ During sepsis and septic shock, the imbalance of oxygen supply and demand can cause microcirculation disturbance and tissue hypoxia. Septic cardiomyopathy has many different manifestations, mainly including left ventricular and or right ventricular dysfunction in the systolic or diastolic period, insufficient cardiac output and oxygen supply, or primary myocardial cell injury, and the disturbance of hemodynamics after cardiac dysfunction is often the initial link of multiple organ failure.^6^

The main pathological characteristics of cardiac insufficiency are the disorder of myocardial structure and function, including myocardial apoptosis, myocardial fibrosis, left ventricular dysfunction, and metabolic disorders.^7^, ^8^ In particular, NLR family pyrin domain containing 3 (NLRP3) in myocardial cells is closely associated with the death of myocardial cells.^9^, ^10^ Under the stimulation of pathological factors, various pathways activate NLRP3 in cardiomyocytes to erupt inflammation cascade, ultimately leading to cardiomyocyte damage.^11^

Existing studies suggest that the activation of NLRP3 inflammatory corpuscles is closely related to multiple organ damage caused by sepsis.^7^, ^8^ NLRP3 inflammatory bodies in cardiovascular diseases, including atherosclerosis, myocardial infarction, pressure load‐induced myocardial remodeling, diabetes cardiomyopathy, and so on. Most studies have shown that NLRP3 is upregulated in myocardial fibrosis induced by various causes and can promote myocardial fibrosis.^9^, ^10^ Inhibition or knockout of NLRP3 can play an antifibrotic role through inflammatory corpuscle‐dependent or noninflammatory corpuscle‐dependent signal pathways. However, NLRP3 is downregulated in myocardial remodeling induced by pressure load of aortic ligation, and NLRP3 knockout can downregulate inflammation signaling pathway, and aggravate cardiac hypertrophy, fibrosis, and inflammatory reaction.

The activation of interleukin‐1β (IL‐1β), the effectors of NLRP3 inflammatory bodies, can trigger the release of other inflammatory mediators, expand the inflammatory response, and directly participate in the progressive destruction of cardiac tissue. Many studies have found that some NLRP3 inhibitors can effectively reduce the occurrence and development of inflammation.^11^ IL‐1β is one key factor of intense inflammation in myocardial infarction. MCC950 selectively inhibits NLRP3, reduces the formation of the shear body, inhibits the maturation of IL‐1 and IL‐18, and reduces the range of infection‐induced cardiac dysfunction (IICD) and fibrosis.^11^

Myo‐inositol oxygenase (MIOX) is a nonheme iron oxygenase, which can catalyze the cleavage reaction of the aliphatic ring. Current research shows that the main function of MIOX is to participate in the metabolism of inositol in tissues.^12^ MIOX can catalyze the oxidative decomposition of inositol to d‐gluconic acid, and further metabolize to xylitol, and this pathway has been proved to be the only pathway of inositol metabolism, more importantly, the reaction of inositol catalyzed by MIOX specifically expressed in renal tissue mainly occurs in the epithelial cells of proximal convoluted renal tubules in the renal cortex.^13^ As mentioned earlier, inositol has a close relationship with a variety of metabolic diseases, suggesting that MIOX may have a close relationship with renal diseases, especially renal tubulointerstitial injury.^14^ Herein, we attempted to determine whether MIOX has proinflammation gene in IICD and its underlying mechanism.

## 2 MATERIALS AND METHODS

### 2.1 Clinical experiments

Patients with IICD (*n* = 26) were collected by our hospital from May 2020 to August 2020. The control group was a normal volunteer (*n* = 26). All procedures performed in studies involving human participants were following the ethical standards of the Independent Ethics Committee for Clinical Research of the Rui Jin Hospital, Lu Wan Branch, Shanghai Jiaotong University School of Medicine (No. 20180817S12). Informed consent was obtained from all individual participants included in the study.

### 2.2 Animals and experimental model and lentivirus injection

IICD mouse model was induced into male db/db mice (7‐week‐old) by cecal ligation and puncture (CLP). The study was approved by the Animal Care and Use Committee of our Hospital (No.2019102111M02) and performed in accordance with the NIH Guidelines for the Care and Use of Laboratory Animals. Six hours later, the myocardial tissues were obtained for further measurement. LV‐MIOX (3.33 × 10^9^ TU/mL) and LV‐control short hairpin RNA (shRNA) (5.26 × 10^9^ TU/mL; Vector) were generated by Genechem. Male db/db mice (*N* = 20) were randomly allocated to two groups: vector (*n* = 10) and sh‐MIOX (*n* = 10) group. All mice were injected with 20 μL of LV‐MIOX or LV‐control shRNA using a 0.5‐mL insulin syringe. On the second day, all mice were induced by CLP. Left ventricular internal diameter, left ventricular ejection fraction, left ventricular fractional shortening, and left ventricular stroke volume were obtained from Nillar pressure‐volume system (MPVS‐400). Then, mice were induced with 50 mg/kg of pentobarbital sodium, caudal vein were collected from and mice were cervical dislocation.

### 2.3 Histological analysis

Tissue samples were fixed in 4% paraformaldehyde, and histological analysis was performed according to Pu et al.^15^, ^16^ Tissue samples were observed using a ﬂuorescence microscope (Zeiss Axio Observer A1).

### 2.4 Cell culture and transfection

H9C2 cell was cultured in Dulbecco's modified Eagle's medium (Gibco; Thermo Fisher Scientific, Inc.) supplemented with 10% fetal bovine serum (Gibco; Thermo Fisher Scientific, Inc.) at 37°C in a humidified atmosphere at 5% CO_2_ in the air. Plasmid DNA (MIOX or NLRP3) or si‐mimics (MIOX or NLRP3) and 5 μL of Lipofectamine 2000 (Invitrogen) were diluted with 250 μL Opti‐MEM, respectively, and incubated for 5 min at room temperature. After 48 h of transfection, cells were induced with 200 ng of lipopolysaccharide (LPS) for 4 h and then cultured with 1 mM of adenosine triphosphate for 30 min.

### 2.5 Quantitative analysis of mRNAs and gene microarray hybridization

Total RNA was extracted using the TRIzol reagent (Life Technologies). Quantitative reverse transcription‐polymerase chain reaction was carried out using an ABI Prism 7900 Sequence detection system (Applied Biosystems). Total RNA was labelled using cyanine‐5‐cTP and hybridized to the SurePrint and G3 Mouse Whole Genome GE 8x60K Microarray G4852A platform. Images were quantified and feature‐extracted using Agilent Feature Extraction Software (Agilent Technologies Inc.).

### 2.6 ELISA

IL‐1β (H002), creatine kinase‐MB (CK‐MB) (H197‐1‐1), creatine kinase (CK) (A032‐1‐1), IL‐6 (H007‐1‐1), and tumor necrosis factor‐α (TNF‐α) (H052‐1) kits were used to measure correlation factors by Enzyme‐linked immunosorbent assay (ELISA) kits (Nanjing Jiancheng Bioengineering Research Institute). Interferon‐γ (PI511 and PI508) kits were used to measure correlation factors by ELISA kits (Beyotime).

### 2.7 Immunofluorescence

Cells were fixed with 4% paraformaldehyde, permeabilized with 0.5% Triton X‐100 in phosphate‐buffered saline for 15 min at room temperature, and blocked with 5% bovine serum albumin for 30 min at 37°C. Cells were treated with primary antibodies MIOX (ab154639, 1:200; Abcam) and NLRP3 (ab4207, 1:200; Abcam) at 4°C overnight: cells were then incubated with conjugated secondary antibodies, stained with DAPI (4′,6‐diamidino‐2‐phenylindole), and observed under a fluorescent illumination microscope (Olympus IX71).

### 2.8 Western blot analysis

Total protein was collected from tissues using RIPA assay on ice. Proteins were separated by sodium dodecyl sulfate‐polyacrylamide gel electrophoresis with 10% separating gels and then transferred to polyvinylidene difluoride membranes. The membranes were blocked in 5% skim milk for 1 h and incubated with primary antibodies against MIOX (ab199729, 1:1000; Abcam), NLRP3 (ab263899, 1:5000; Abcam), and β‐actin (ab8226, 1:5000; Abcam) at 4°C overnight. Membranes were then incubated with secondary antibodies (1:5000) for 2 h. Enhanced Chemiluminescence (ECL) Plus kit (Millipore) was applied for visualization and protein bands were calculated using Image J software.

### 2.9 Statistical analysis

Data are presented as means ± standard deviations (reported number = 3 or 6 or 26) using SPSS 23.0 (SPSS). Student's *t*‐test or one‐way analysis of variance was used as appropriate. *p* < .05 was considered to represent the statistical significance.

## 3 RESULTS

### 3.1 Infection‐induced MIOX expression level in the model of IICD

**Figure 1:**
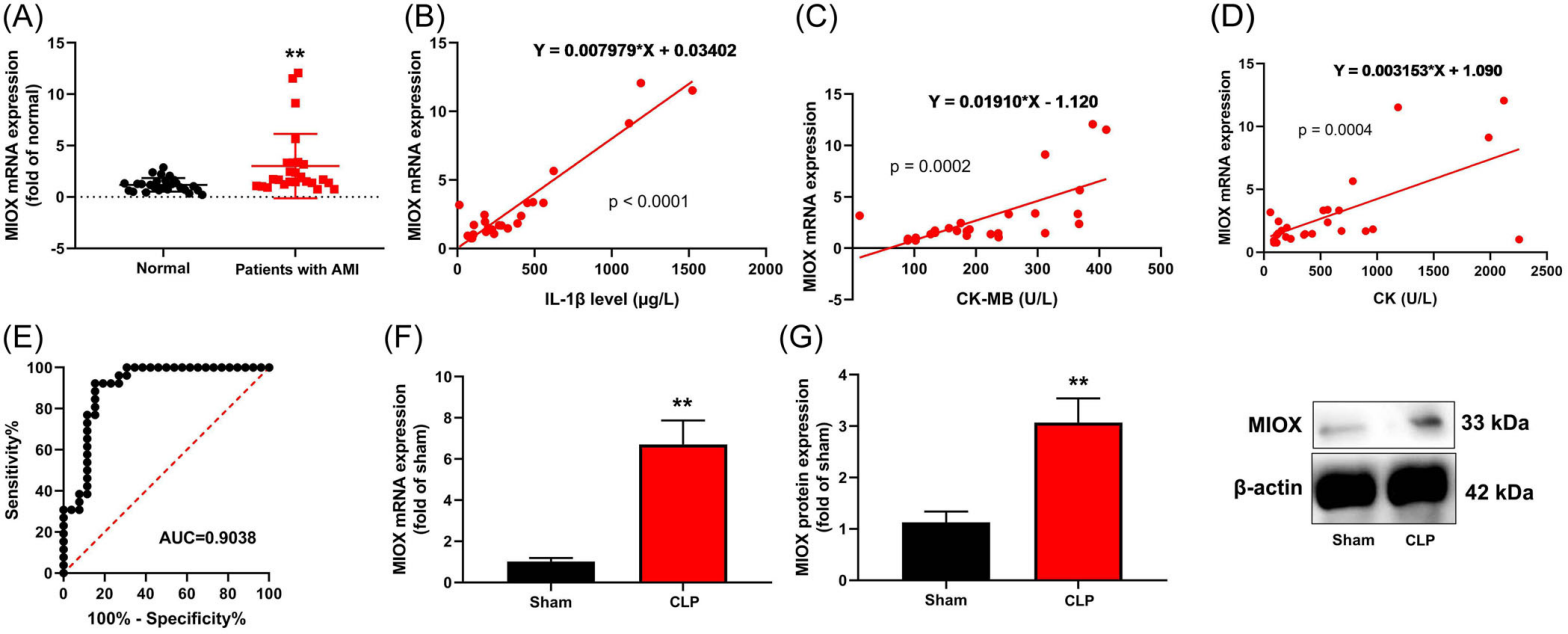
Infection‐induced Myo‐inositol oxygenase (MIOX) expression level in the model of cardiac dysfunction. MIOX messenger RNA (mRNA) expression level (A), serum MIOX mRNA levels was a positive correlation with serum interleukin‐1β (IL‐1β) levels (B), creatine kinase‐MB (CK‐MB) (C) or CK (D) levels, the receiver‐operating characteristic (ROC, E) in patients with infection‐induced cardiac dysfunction; MIOX mRNA (F) and protein (G) expression level in mice with infection‐induced cardiac dysfunction. Number of patients = 26; number of mice = 6. Normal, normal volunteer group; Model, patients with infection‐induced cardiac dysfunction group; Sham, sham control group; AMI, mice with myocardial infarction group; **p < .01 compared with a normal volunteer group or sham control group. CLP, cecal ligation and puncture.

First, the serum expression of MIOX messenger RNA (mRNA) level in patients with IICD was upregulated, as compared with normal healthy volunteers (Figure 1A). Additionally, serum MIOX mRNA levels were a positive correlation with serum IL‐1β levels, CK‐MB, or CK levels in patients with IICD and the receiver‐operating characteristic (ROC) curve was constructed to assess the diagnostic value of MIOX level (Figure 1B–E).

Next, in infection‐induced mice with cardiac dysfunction, MIOX protein and mRNA expressions in heart tissue were induced (Figure 1F,G).

### 3.2 MIOX gene promoted inflammation in the in vitro model of IICD

**Figure 2:**
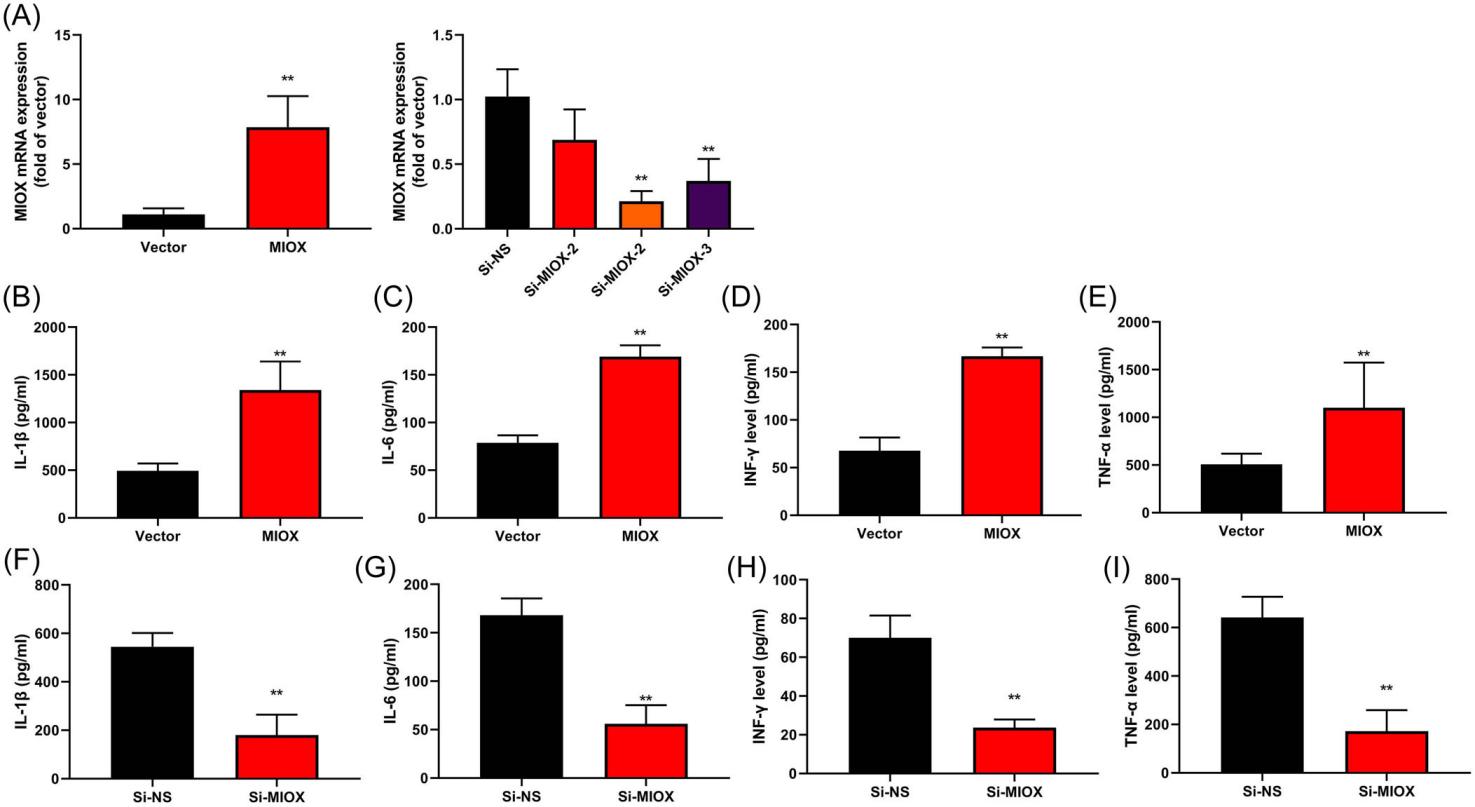
Myo‐inositol oxygenase (MIOX) gene promoted inflammation in the in vitro model of infection‐induced cardiac dysfunction. MIOX messenger RNA (mRNA) expression in the in vitro model (A); interleukin‐1β (IL‐1β) (B), IL‐6 (C), interferon‐γ (INF‐γ) (D), and tumor necrosis factorF‐α (TNF‐α) (E) levels in the in vitro model by overexpression of the MIOX group; IL‐1β (F), IL‐6 (G), INF‐γ (H), and TNF‐α (I) levels in the in vitro model by downregulation of the MIOX group. Number of vitro model = 3. Vector, control negative group; MIOX, overexpression of the MIOX group; si‐NS, si‐negative group; si‐MIOX, downregulation of the MIOX group. **p < .01 compared with the control negative group or si‐negative group.

This experiment investigated the function of MIOX on inflammation in the in vitro model of IICD. MIOX mRNA expression was increased in the MIOX group and MIOX mRNA expression was downregulated in si‐MIOX group (Figure 2A). MIOX promoted inflammation levels in the in vitro model of IICD (Figure 2B–E). Downregulation of MIOX inhibited inflammation levels in the in vitro model of IICD (Figure 2F–I).

### 3.3 MIOX accelerated inflammation and cardiac dysfunction in infection‐induced mice

**Figure 3:**
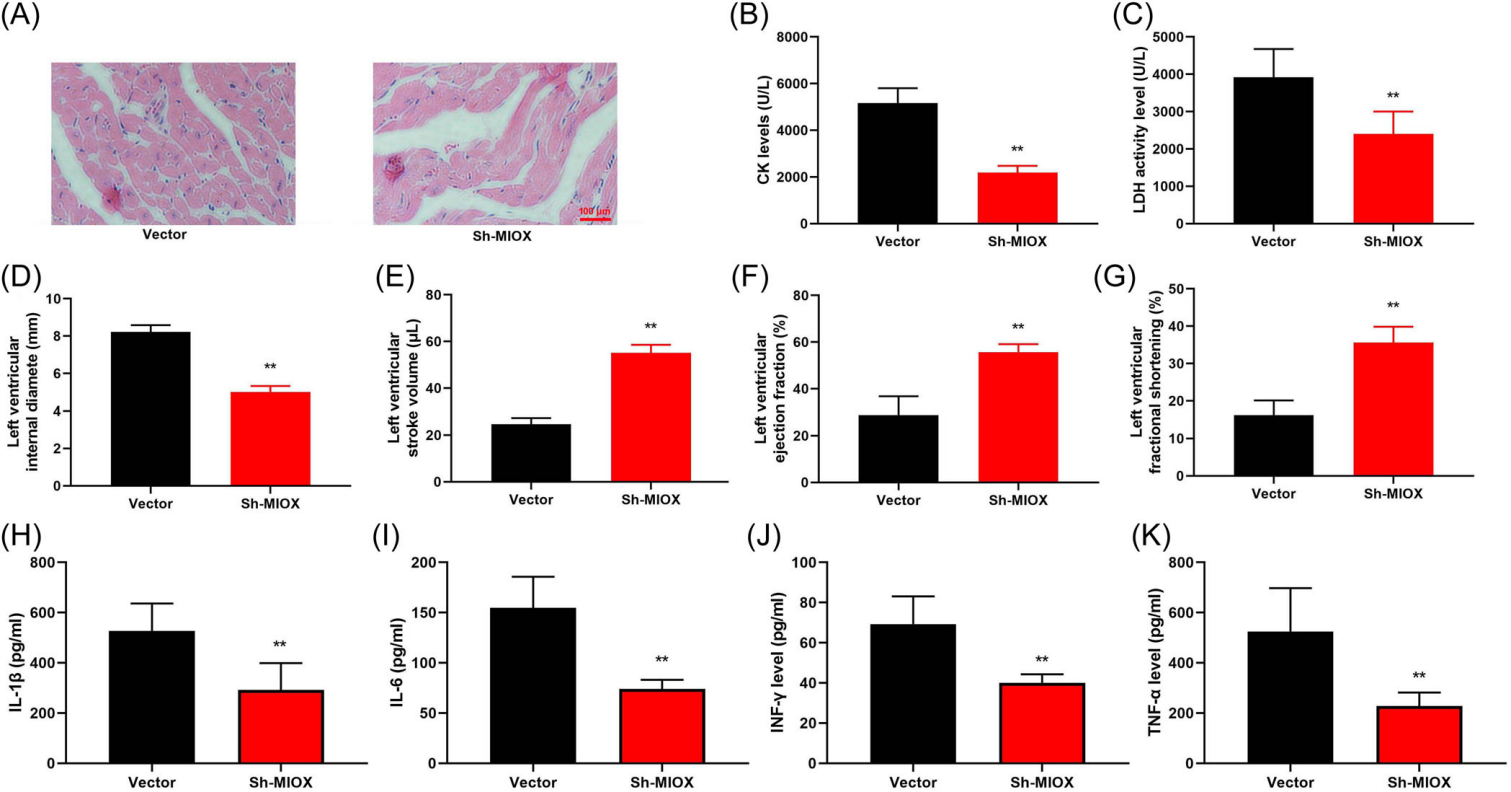
Myo‐inositol oxygenase (MIOX) accelerated inflammation and cardiac dysfunction in infection‐induced mice. Myocardium (A), creatine kinase (CK) activity level (B), lactate dehydrogenase (LDH) activity level (C), left ventricular internal diameter (D), left ventricular stroke volume (E), left ventricular ejection fraction (F), left ventricular fractional shortening (G), interleukin‐1β (IL‐1β) (H), interleukin‐6 (IL‐6) (I), interferon‐γ (INF‐γ) (J), and tumor necrosis factor‐α (TNF‐α) (K) levels in mice with infection‐induced cardiac dysfunction. Number of mice = 6. Vector, mice with infected‐induced cardiac dysfunction by vector; sh‐MIOX, mice with infected‐induced cardiac dysfunction by sh‐MIOX. **p < 0.01 compared with vector group.

We examined that the function of MIOX in an infection‐induced mice model of cardiac dysfunction. Sh‐MIOX lessened myocardial fibrosis, reduced CK level and lactate dehydrogenase activity level, decreased myocardial infarction, reduced left ventricular ejection fraction, left ventricular fractional shortening and left ventricular stroke volume, increased left ventricular internal diameter, and suppressed inflammation levels in the infection‐induced mice model of cardiac dysfunction (Figure 3).

### 3.4 MIOX interacted with NLRP3 protein to reduce the degradation of NLRP3

**Figure 4:**
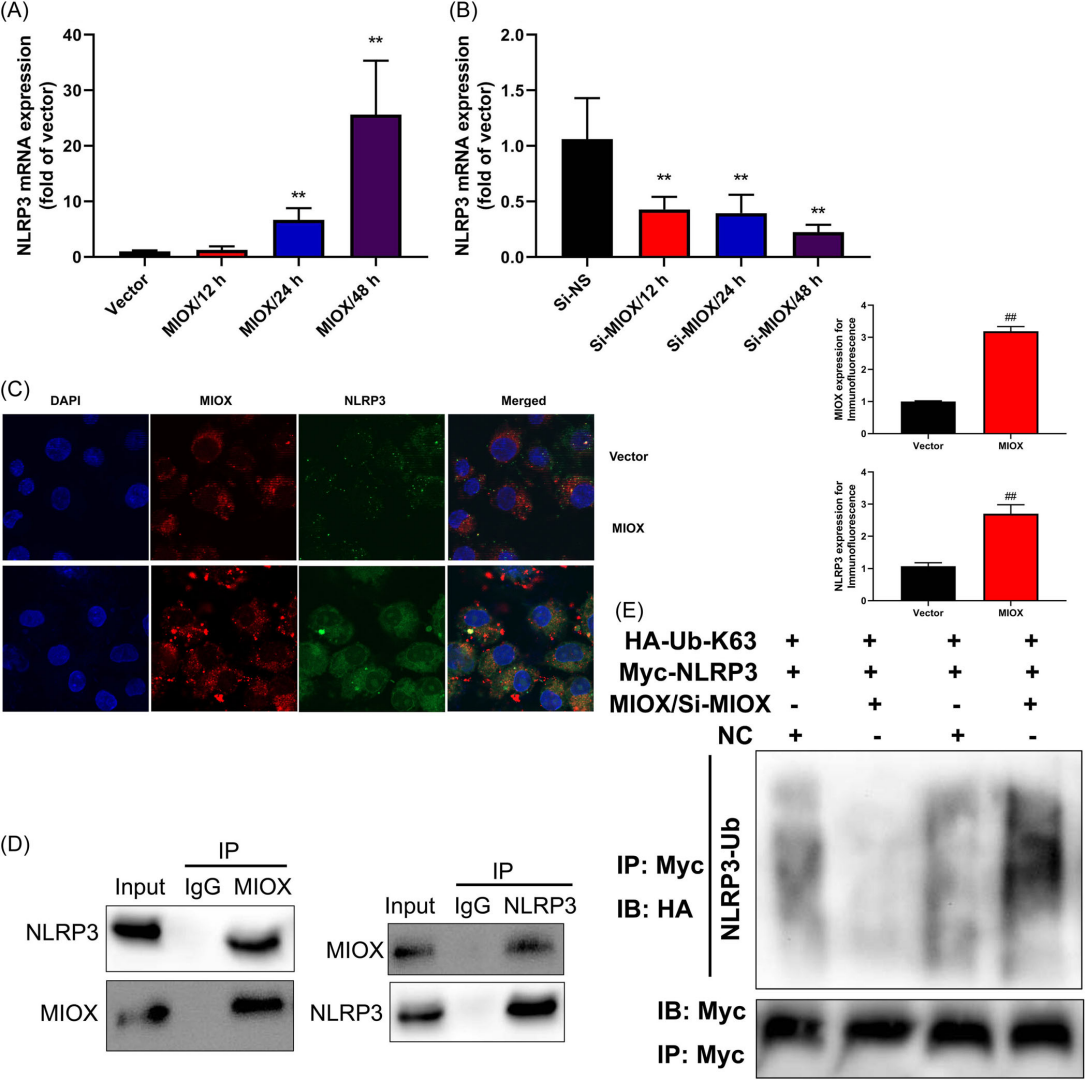
Myo‐inositol oxygenase (MIOX) interacted with the NLR family pyrin domain containing 3 (NLRP3) protein to reduce the degradation of NLRP3. NLRP3 mRNA expression in the in vitro model by overexpression of MIOX (A) and downregulation of MIOX (B); NLRP3 and MIOX expression in the in vitro model (immunofluorescence; C), NLRP3 and MIOX (immunopreciptiaton [IP]; D), ubiquitination of NLRP3 (E). Number of vitro model = 3. Vector, control negative group; MIOX, overexpression of MIOX group; si‐NS, si‐negative group; si‐ MIOX, downregulation of MIOX group. **p < .01 compared with the control negative group or si‐negative group. DAPI, 4′,6‐diamidino‐2‐phenylindole; IB, immunblotting;  IP, immunoprecipitation; mRNA, messenger RNA.

The experiment examined the mechanism of MIOX in the model of IICD. MIOX induced NLRP3 mRNA expression in the in vitro model at times 24 and 48 h (Figure 4A). Si‐MIOX suppressed NLRP3 mRNA expression in the in vitro model at times 24 and 48 h (Figure 4B). Overexpression of MIOX increased MIOX and NLRP3 expression in the in vitro model (immunofluorescence; Figure 4C). The association between MIOX and NLRP3 proteins by coimmunoprecipitation (Figure 4D). MIOX reduced the ubiquitination of NLRP3 and si‐MIOX increased the ubiquitination of NLRP3 in the in vitro model (Figure 4E).

**Figure 5:**
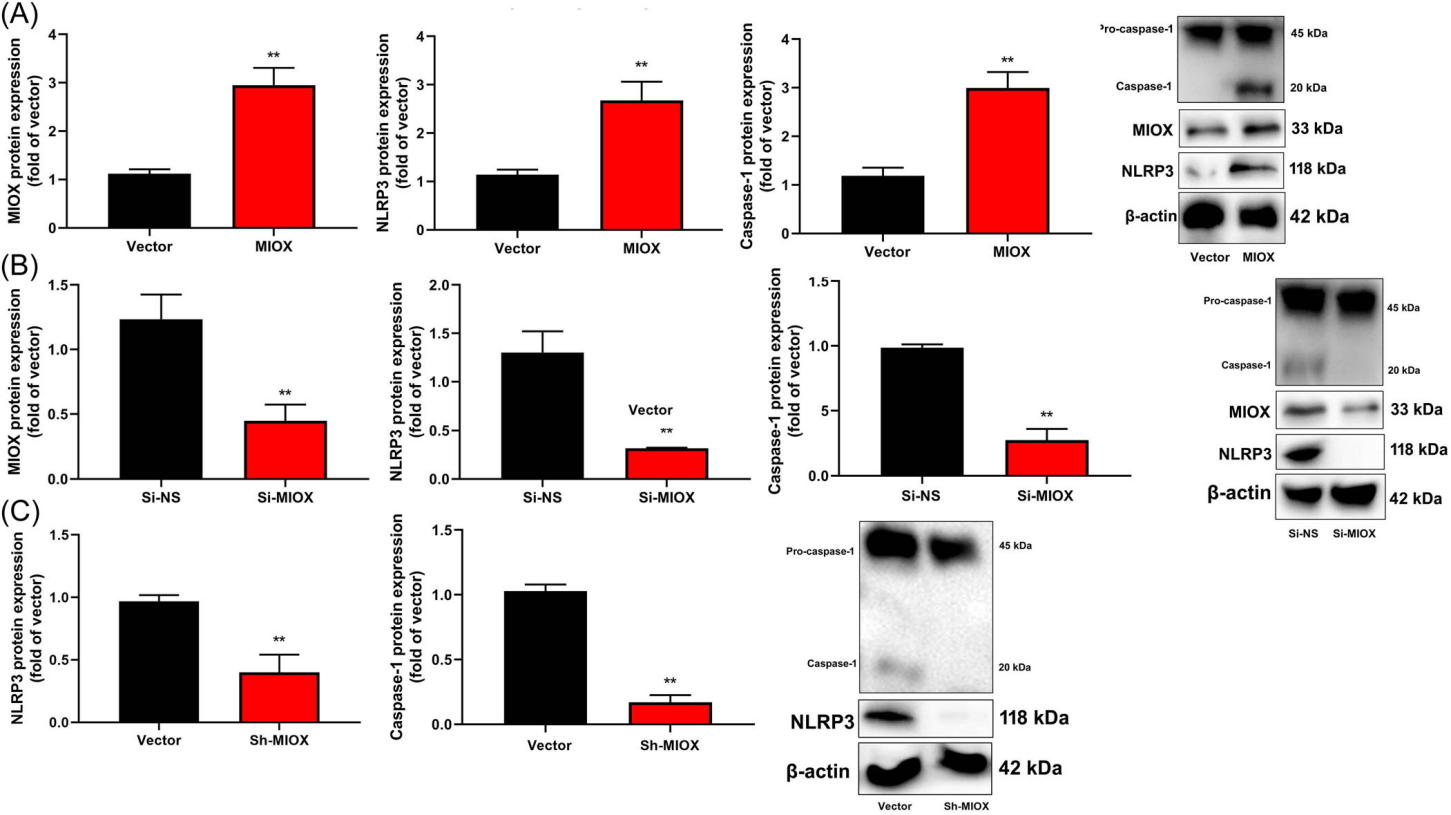
Myo‐inositol oxygenase (MIOX) induced NLR family pyrin domain containing 3 (NLRP3) protein in the model of infection‐induced cardiac dysfunction. MIOX and NLRP3 protein expression in the in vitro model of overexpression of MIOX (A and B); MIOX and NLRP3 protein expression in the in vitro model of downregulation of MIOX (C and D); NLRP3 protein expression in mice with infection‐induced cardiac dysfunction by sh‐MIOX (E). Number of vitro model = 3. Vector, control negative group; MIOX, overexpression of MIOX group; si‐NS, si‐negative group; si‐MIOX, downregulation of MIOX group. **p < .01 compared with the control negative group or si‐negative group. **p < 0.01 compared with vector group or si‐nc group.

Next, MIOX induced MIOX and NLRP3 protein expression in vitro model (Figure 5A,B). Si‐MIOX suppressed MIOX and NLRP3 protein expression in the in vitro model (Figure 5C,D). Sh‐MIOX suppressed NLRP3 protein expression in infection‐induced mice with cardiac dysfunction (Figure 5E).

### 3.5 Inhibition of MIOX reversed the effects of NLRP3 in the in vitro mode of cardiac dysfunction

**Figure 6:**
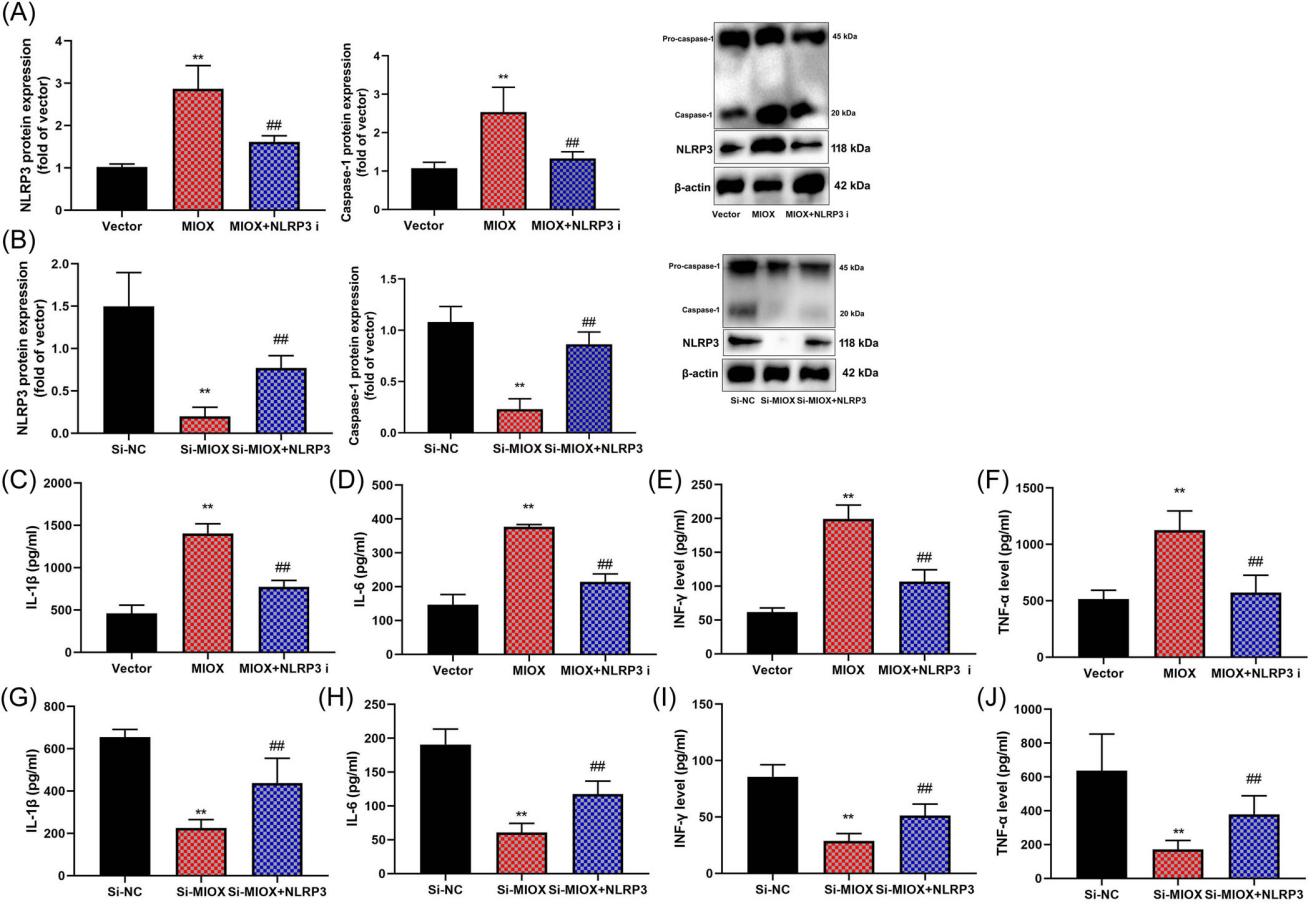
Inhibition of Myo‐inositol oxygenase (MIOX) reversed the effects of NLR family pyrin domain containing 3 (NLRP3) in the in vitro mode of cardiac dysfunction. NLRP3 protein expression in the in vitro model by overexpression of MIOX and downregulation of NLRP3 (A); NLRP3 protein expression in the in vitro model by downregulation of MIOX and overexpression of NLRP3 (B); interleukin‐1β (IL‐1β) (C), IL‐6 (D), interferon‐γ (INF‐γ) (E), and tumor necris factor‐α (TNF‐α) (F) levels in the in vitro model by overexpression of MIOX and downregulation of NLRP3; IL‐1β (G), IL‐6 (H), INF‐γ (I) and TNF‐α (J) levels in the in vitro model by downregulation of MIOX and overexpression of NLRP3. Number of vitro model = 3. Vector, control negative group; MIOX, overexpression of MIOX group; MIOX + si‐ NLRP3, overexpression of MIOX and downregulation of NLRP3 group; si‐NS, si‐negative group; si‐MIOX, downregulation of the MIOX group; si‐MIOX, downregulation of the MIOX group and overexpression of NLRP3. ^##^ *p* < .01 compared with the control negative group or si‐negative group; **p < .01 compared with overexpression of the MIOX group or downregulation of the MIOX group.

To understand the mechanism of MIOX in the infection‐induced model of cardiac dysfunction, si‐NLRP3 suppressed NLRP3 protein expression in the in vitro model of IICD by MIOX (Figure 6A). NLRP3 induced NLRP3 protein expression in the in vitro model of IICD by downregulation of ring finger protein 20 (RNF20) (Figure 6B). Si‐NLRP3 reduced inflammation levels in the in vitro model of IICD by MIOX (Figure 6C–F). NLRP3 increased inflammation levels in the in vitro model of IICD by downregulation of RNF20 (Figure 6G–J).

## 4 DISCUSSION

**Figure 7:**
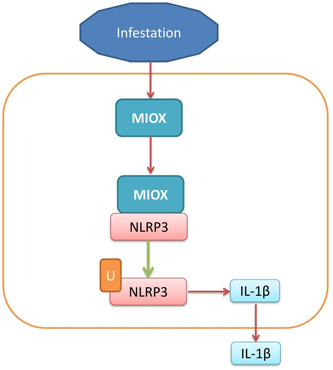
Myo‐inositol oxygenase (MIOX) accelerated inflammation in the model of infection‐induced cardiac dysfunction by NLR family pyrin domain containing 3 (NLRP3) inflammasome. IL, interleukin.

Sepsis has caused a heavy burden on the country, society, and family, and has seriously threatened the health and well‐being of the people.^17^ In 2020, the incidence rate of sepsis in intensive care units in China will be 20.6%, down from the incidence rate in 2014, but the 90‐day mortality rate will still be as high as 35.5%.^18^ Infection is a type of severe organ insufficiency caused by the uncontrolled host‐to‐infection immune response. Although the mortality rate of infection has dropped from 37% to 30.8% after the release of guidelines to rescue infections, further reductions are still problematic.^17^, ^18^ In the past five decades, more than 50% of infected patients have varying degrees of cardiac insufficiency, but the clinical manifestations of sepsis‐related cardiac insufficiency are still not precisely defined.^19^ A large amount of catecholamines are released and hemodynamics are disordered, resulting in tissue ischemia and hypoxia, and lactic acid formation.^18^ When the oxidative stress state inhibits the host's endogenous antioxidant defence system sepsis, a large amount of endotoxin and inflammatory mediators can damage the mitochondrial structure, the body's oxidation/antioxidant system is out of balance, inhibiting the activity of antioxidant enzymes, and producing a large number of ROS, causing oxidative stress. In the early stage of sepsis, ROS and reactive nitrogen species (RNS) were produced and released in large quantities by cells, and the mitochondria were subject to oxidation/nitrification stress.^18^ A large number of ROS,^19^ inorganic phosphate, and RNS mediate the structural protein conformation changes on mitochondrial damage, resulting in the pathological opening of 1‐methyl‐4‐phenyl‐1,2,5,6‐tetrahydropyridine and the increase of mitochondrial permeability, which will not only cause the swelling and destruction of mitochondrial structure but also activate the downstream apoptosis pathway, leading to cardiomyocyte apoptosis. Animal experiments showed that esmolol could increase the activity of catalase and inhibit the oxidative stress reaction of myocardial cells.^19^ The serum expression of MIOX mRNA level in patients with IICD was upregulated, as compared with normal healthy volunteers. Sommese et al.^20^ showed that MIOX led to an overexpression of ROS involved in coronary heart disease. Thus, these results indicated that MIOX exerts a pathogenic factor for IICD. In this study, we only analyzed the expression of MIOX in patients with infection‐induced cardiac dysfunction and normal volunteer (Figure 7). We analyzed the MIOX expression in patients with cardiac dysfunction but not the infection. It is one insufficient for this study, and we should analyze the expression of MIOX in patients with infection‐induced cardiac dysfunction versus MIOX expression in patients with cardiac dysfunction not infection.

Sepsis is the body's complicated immune response to infection and is also one of the main causes of death of critically ill patients in clinical practice.^21^ The main complication of sepsis is cardiac insufficiency.^22^, ^23^ Under the action of chemokines, circulating monocytes are recruited into the myocardial interstitium and are differentiated into macrophages under the action of macrophage colony‐stimulating factor, which in turn leads to increased inflammation in the myocardium, increased vascular leakage, myocardial edema, and affects myocardial compliance, as well as myocardial function.^24^ Our experiment showed that MIOX gene promoted inflammation in the in vitro model of IICD. MIOX accelerated inflammation and cardiac dysfunction in infection‐induced mice. Mertoglu et al.^25^ demonstrated that MIOX is involved in the development of acute kidney injury. These data demonstrated that MIOX accelerated inflammation in the model of IICD.

Studies have found NLRP3 inflammatory corpuscles as one of the targets for the treatment of sepsis. IICD is a disease accompanied by an inflammatory reaction, such as the infiltration of inflammatory cells and the release of inflammatory cytokines/chemokines, leading to myocardial injury and remodeling, affecting the patient's condition and mortality.^23^ NLRP3 inflammatory corpuscles was significantly increased in ischemic myocardial fibroblasts of rats and mice with permanent IICD. The upregulation of NLRP3 expression in ischemic myocardium can promote the expression of caspase‐1 and IL‐1b, and induce the cascade reaction of myocarditis. Inhibiting the expression of NLRP3 inflammatory body can reduce inflammation and improve cardiac dysfunction and remodeling in rats with IICD.^23^ Intervention of abnormal expression of NLRP3 inflammatory bodies may become a new treatment of IICD.^23^ The persistent chronic inflammation of the myocardium may be associated with myocardial hypertrophy, fibrosis, and cardiac insufficiency, which can promote the occurrence and development of diabetic cardiomyopathy.^15^, ^23^, ^26^, ^27^ Consistent with these findings, we found that MIOX interacted with NLRP3 protein to reduce the degradation of NLRP3. The suppression of MIOX reversed the effects of NLRP3 in the in vitro model of IICD. These results suggested that MIOX could activate the NLRP3 inflammasome in the model of IICD. In this study, we used the CLP‐induced mice model; this model might relatively severe inflammation model, and we will used LPS or other induction modes into mice model in further experiments.

In summary, our research clarified that MIOX accelerated inflammation in the model of IICD by NLRP3 inflammasome. MIOX may represent a promising inflammation factor for the treatment of IICD.

## AUTHOR CONTRIBUTIONS

Material preparation, data collection, and analysis were performed by Wenjun Zhou and Congyi Yu. The first draft of the manuscript was written by Congyi Yu and all authors commented on previous versions of the manuscript. All authors read and approved the final manuscript.

## CONFLICT OF INTEREST STATEMENT

The authors declare no conflict of interest.

## ETHICS STATEMENT

All procedures performed in studies involving human participants were following the ethical standards of the Independent Ethics Committee for Clinical Research and Animal trials of the Rui Jin Hospital, Lu Wan Brance, Shanghai Jiaotong University School of Medicine (Nos. 20180817S12 and 2019102111M02). Informed consent was obtained from all individual participants included in the study. This manuscript does not contain personal and/or medical information about an identifiable living individual.

## Data Availability

The data sets used and/or analyzed during the current study are available from the corresponding author on reasonable request.
